# Effect of Semi-sitting Position on Airway Clearance Obtained by Jaw Thrust During Oral Fiberoptic-Guided Intubation

**DOI:** 10.7759/cureus.109464

**Published:** 2026-05-22

**Authors:** Kumar Saurabh, Yasana Shami

**Affiliations:** 1 Anesthesiology and Critical Care, Maulana Azad Medical College, New Delhi, IND; 2 Anesthesiology and Critical Care, Lady Hardinge Medical College, New Delhi, IND

**Keywords:** airway clearance, anesthesia airway management, epiglottis visualization, fiberoptic intubation, jaw thrust, semi-sitting position, soft palate obstruction

## Abstract

Background: Oral fiberoptic-guided intubation can be challenging in anesthetized patients due to loss of pharyngeal muscle tone, resulting in posterior displacement of the tongue, soft palate, and epiglottis. Maneuvers such as jaw thrust can improve patency, but their effectiveness may vary with patient position. Semi-sitting positioning has been shown to reduce upper airway collapsibility. This study compared airway clearance obtained by jaw thrust in the semi-sitting versus supine position during oral fiberoptic intubation.

Methods: This prospective randomized comparative study included adult American Society of Anesthesiologists (ASA) I-II patients aged 18-60 years undergoing elective surgery with orotracheal intubation. Patients were randomized into two groups: semi-sitting at 25° with jaw thrust (Group SS) and supine with jaw thrust (Group S). Airway clearance at the soft palate level (primary outcome) and at the epiglottis, time to visualize the vocal cords and carina, attempts required for tube advancement, total intubation time, and post-extubation airway trauma were assessed. Statistical analysis used chi-square and Student's t-test, with p<0.05 considered significant.

Results: Sixty patients completed the study (30 per group). Airway clearance at the soft palate was significantly better in the semi-sitting group (p<0.001). Clearance at the epiglottis was comparable between groups (p=0.100). Time to visualize the vocal cords (12.35±6.68 vs. 15.99±5.37 s; p=0.024) and carina (19.92±7.10 vs. 25.05±7.03 s; p=0.007) was significantly shorter in the semi-sitting group. Tube advancement attempts, time for tube passage, total intubation time, and incidence of trauma or postoperative sore throat were similar across groups. No intubation failures or desaturation occurred.

Conclusion: Jaw thrust in the 25° semi-sitting position provides superior airway clearance at the soft palate and allows faster visualization of the vocal cords and carina during oral fiberoptic intubation. Overall intubation time and tube advancement characteristics remain similar between positions. Semi-sitting positioning may therefore be a useful adjunct to improve pharyngeal patency and facilitate fiberoptic-guided intubation in anesthetized patients.

## Introduction

Fiberoptic-guided intubation remains an important airway management technique, particularly when conventional direct laryngoscopy is anticipated to be difficult or undesirable [1,2]. Successful bronchoscopic intubation requires adequate maintenance of upper airway patency throughout the procedure. During general anesthesia, reduced pharyngeal muscle tone and altered airway mechanics may promote airway narrowing and impair visualization of laryngeal structures [3-5].

Various maneuvers have been used to improve airway patency during fiberoptic-guided intubation, including mandibular advancement, lingual traction, airway adjuncts, and positional modifications [6-10]. Among these, the jaw thrust maneuver is simple and widely practiced because anterior displacement of the mandible may enlarge the pharyngeal airway space [7,8]. However, the effectiveness of jaw thrust may be influenced by patient positioning.

Although airway adjuncts such as the Berman and Ovassapian intubating airways may facilitate glottic visualization and insertion of the fiberscope during oral fiberoptic-guided intubation, they may also interfere with advancement of the endotracheal tube by obstructing tube passage or catching the cuff of the tube during railroading. In addition, these airway adjuncts may not be readily available in all clinical settings [6,10].

Body position affects upper airway configuration during anesthesia. Head-up or semi-sitting positioning has been associated with reduced upper airway narrowing and improved laryngeal visualization compared with the supine position [11-13]. Combining jaw thrust with semi-sitting positioning may therefore further improve airway clearance during fiberoptic-guided intubation.

Although previous studies have evaluated airway patency in different patient positions, evidence directly comparing airway clearance achieved with jaw thrust in semi-sitting and supine positions during oral fiberoptic-guided intubation remains limited [13]. Therefore, we aimed to compare soft palate-level airway clearance achieved with jaw thrust in the semi-sitting versus supine position during oral fiberoptic-guided intubation. Secondary outcomes included epiglottic-level airway clearance, time to visualization of the vocal cords and carina, number of attempts required for successful tube advancement, total intubation time, and airway-related complications, including trauma, sore throat, and hoarseness.

## Materials and methods

Study design and duration

This prospective randomized comparative study was conducted in the Department of Anesthesiology and Intensive Care at Maulana Azad Medical College and the associated Lok Nayak Hospital, New Delhi, over 30 months from September 2015 to March 2018, after obtaining approval from the Institutional Ethics Committee.

Study population and eligibility criteria

Sixty adult patients of either sex, aged 18-60 years, with American Society of Anesthesiologists (ASA) physical status I or II, scheduled for elective surgical procedures under general anesthesia requiring oral endotracheal intubation were included. Written informed consent was obtained from all participants. Patients with anticipated difficult airway, known bleeding or coagulation disorders, recent upper respiratory tract infection, and those planned for awake or nasal intubation were excluded.

Sample size and randomization

Considering airway clearance at the soft palate as the primary outcome, previous studies reported clearance rates of 72.7% in the supine position and 95.5% in the semi-sitting position [13]. Based on an alpha error of 5% and statistical power of 80%, the calculated sample size was 69 patients in each group. However, as this was a time-bound study, a total of 60 patients were included, with 30 patients in each group. Patients were randomly allocated into two groups using a computer-generated randomization table: Group SS (semi-sitting position) and Group S (supine position).

Operator criteria

The jaw thrust maneuver and fiberoptic-guided intubation were performed by anesthesiologists regularly practicing fiberoptic intubation, each having performed at least 10 successful oral fiberoptic-guided intubations in manikins and 20 in adult patients. In all cases, the jaw thrust maneuver was applied by a trained anesthesiologist.

Anesthesia technique and procedure

All patients were kept fasting as per standard guidelines. Premedication included oral alprazolam 0.5 mg on the night before surgery and intramuscular glycopyrrolate 0.2 mg administered 30 minutes before induction. Standard monitoring with electrocardiography, noninvasive blood pressure, and pulse oximetry was instituted, and baseline parameters were recorded.

Anesthesia was induced with intravenous fentanyl 2 µg/kg and propofol 2 mg/kg, followed by neuromuscular blockade with vecuronium 0.1 mg/kg. Ventilation was maintained using oxygen and isoflurane via face mask.

**Figure 1:**
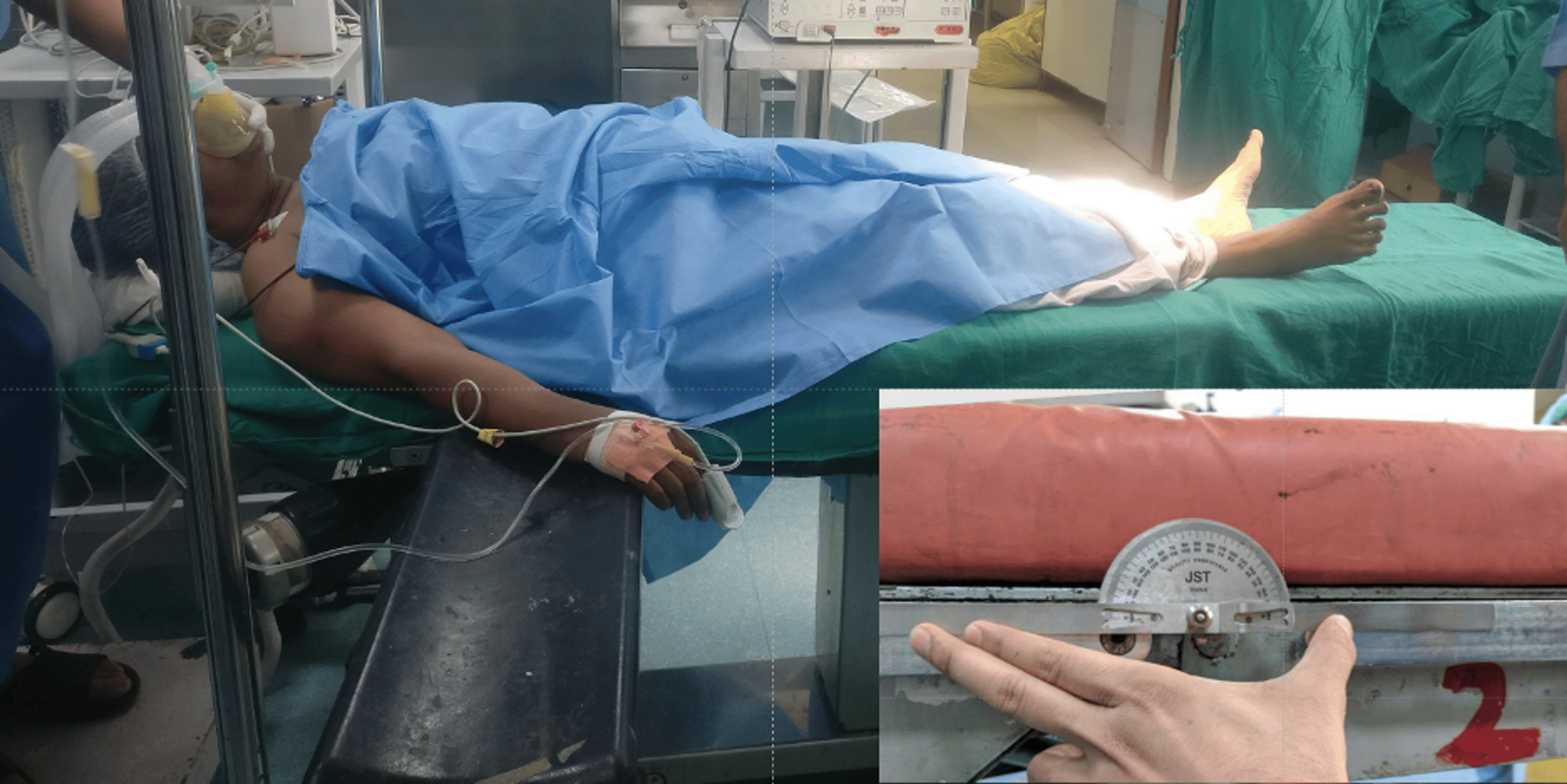
Supine (horizontal) position The patient is positioned supine on the operating table with the head in a neutral position. The horizontal alignment of the table is confirmed using a goniometer. This position was used in Group S during fibreoptic-guided intubation.

In the supine group (Group S), patients were maintained in a horizontal position with the head in neutral alignment during fiberoptic-guided intubation (Figure 1).

**Figure 2:**
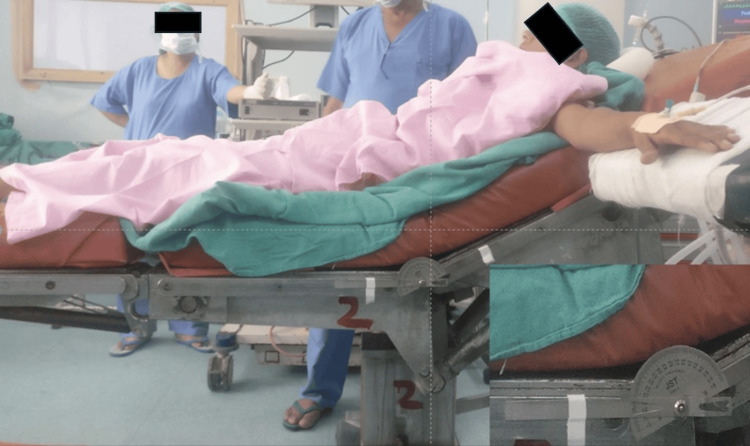
Semi-sitting (25° back-up) position The patient is positioned in a 25° head-up (back-up) position achieved by adjusting the operating table configuration. The angle is confirmed using a goniometer. This position was used in Group SS during fiberoptic-guided intubation.

In the semi-sitting group (Group SS), a 25° head-up position was achieved by adjusting the operating table, and the angle was confirmed using a goniometer before fiberoptic-guided intubation (Figure 2).

**Figure 3:**
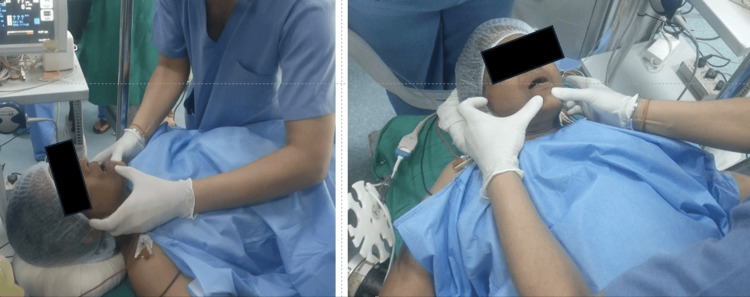
Application of the jaw thrust maneuver

Oral fiberoptic-guided intubation was performed by an experienced anesthesiologist. A trained assistant applied the jaw thrust maneuver in all cases. The assistant stood facing the patient from the left side, with the thumbs used to open the mouth and the fingers placed behind the ramus of the mandible, applying an upward and anterior thrust (Figure 3).

**Figure 4:**
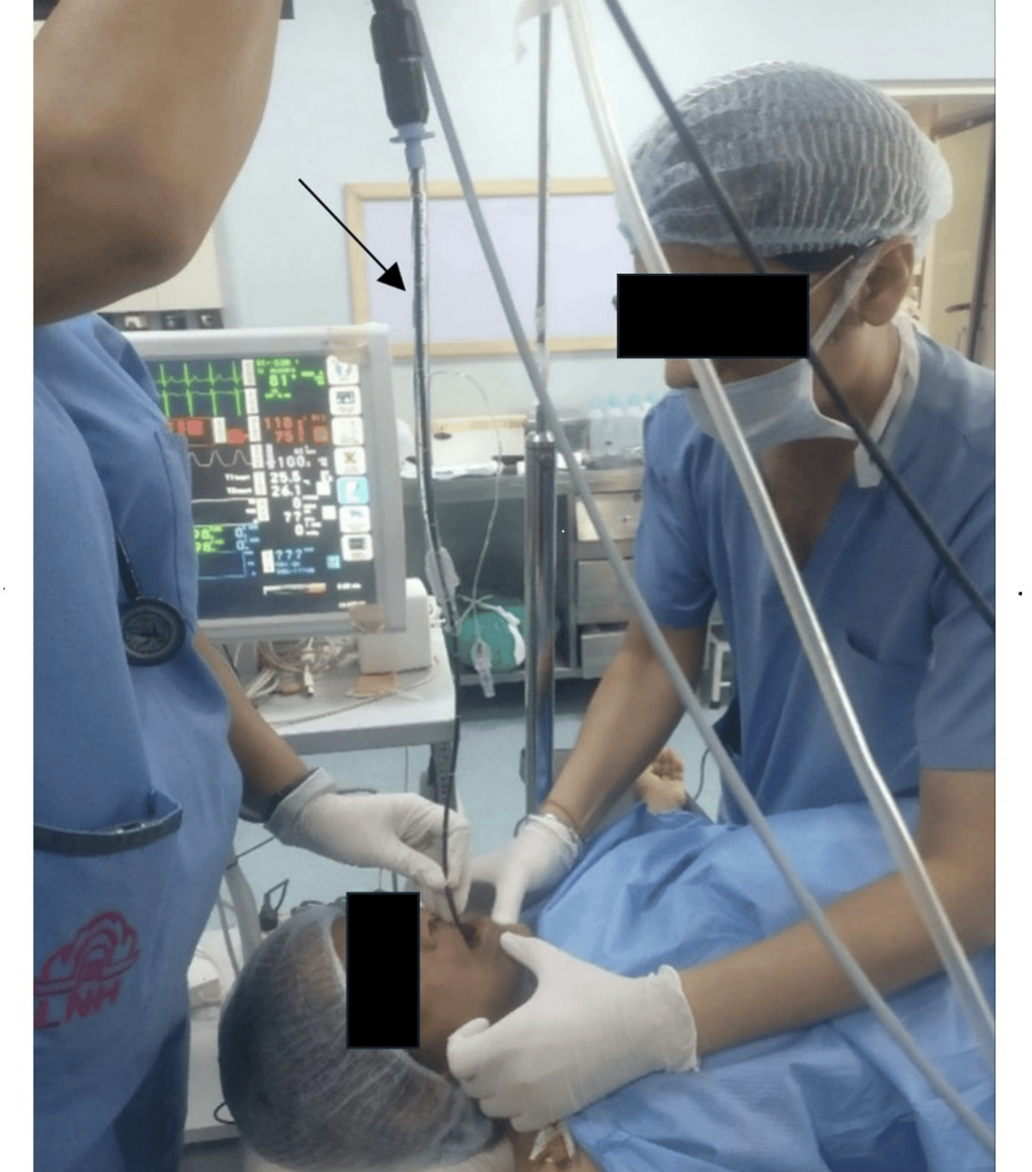
Oral fibreoptic-guided intubation with jaw thrust maneuver

An appropriately sized endotracheal tube (8.5 mm internal diameter for males and 7.5 mm for females) was preloaded over the fiberoscope, with the concave curvature oriented anteriorly and the bevel directed toward the patient’s left side (Figure 4). The fiberoscope was advanced orally along the dorsum of the tongue. Oxygen supplementation was provided via nasal prongs throughout the procedure.

If visualization of the vocal cords and carina was not achieved within 60 seconds or oxygen saturation decreased below 92%, mask ventilation was resumed before reattempting. A maximum of three attempts was allowed, after which intubation was performed using direct laryngoscopy if required.

If resistance was encountered during advancement of the endotracheal tube, it was withdrawn slightly and rotated 90° counterclockwise before re-advancement.

Primary outcome

**Figure 5:**
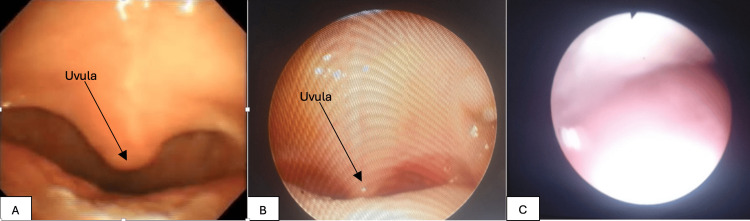
Fiberoptic views demonstrating airway clearance at the soft palate level Fiberoptic views showing grading of airway clearance at the soft palate level: (A) no obstruction, where the uvula does not touch the dorsum of the tongue; (B) partial obstruction, where the uvula or part of the soft palate is in contact with the tongue; and (C) complete obstruction, where the soft palate is fully apposed to the dorsum of the tongue.

The primary outcome was airway clearance at the soft palate level, assessed by observing the relationship between the uvula or soft palate and the dorsum of the tongue (no obstruction, partial obstruction, or complete obstruction), as demonstrated in Figure 5.

Secondary outcome

**Figure 6:**
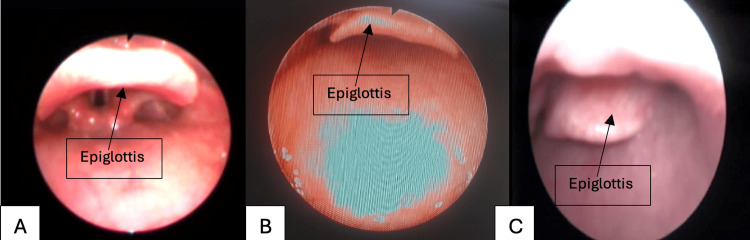
Fiberoptic views demonstrating airway clearance at the epiglottis level Fiberoptic views showing grading of airway clearance at the epiglottis level: (A) no obstruction, where the epiglottis does not contact the posterior pharyngeal wall; (B) partial obstruction, where the epiglottis is partially in contact with the posterior pharyngeal wall; and (C) complete obstruction, where the epiglottis is fully apposed to the posterior pharyngeal wall.

The secondary outcome included airway clearance at the epiglottis level, assessed according to the degree of contact between the epiglottis and the posterior pharyngeal wall (no obstruction, partial obstruction, or complete obstruction), as demonstrated in Figure 6.

Other secondary outcome parameters included time to visualization of the vocal cords, time to visualization of the carina, time for endotracheal tube advancement, number of attempts required for successful intubation, and total intubation time.

Airway trauma was assessed by the presence of blood staining on the endotracheal tube at extubation, as well as the incidence of postoperative sore throat or hoarseness at one and six hours after extubation.

Statistical analysis

Statistical analyses were performed using the latest version of Statistical Package for the Social Sciences (SPSS) software. Continuous variables were expressed as mean±standard deviation and analyzed using Student's t-test. Categorical variables were expressed as frequencies and percentages and analyzed using the chi-square test. Fisher’s exact test was applied when the expected cell frequency was less than five. A p-value of <0.05 was considered statistically significant.

## Results

**Figure 7:**
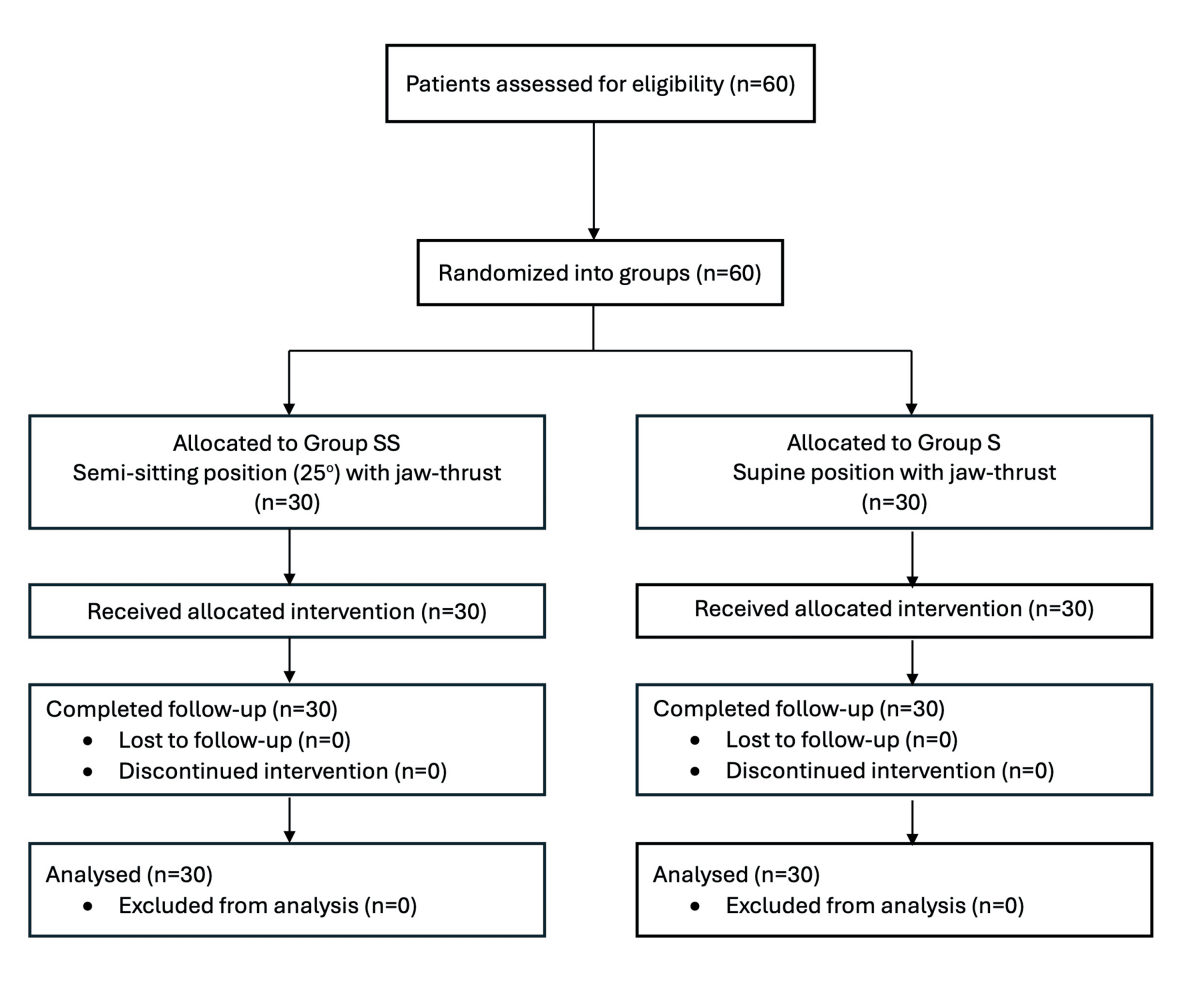
Flow diagram of patient recruitment, allocation, follow-up, and analysis

The present study was conducted on 60 consenting patients aged between 18 and 60 years who were randomly allocated to one of two groups, Group SS and Group S, comprising 30 patients each (Figure 7).

As shown in Table 1, airway clearance at the soft palate level was significantly better in the SS group than in the S group, with a p-value <0.001.

**Table 1:** Airway clearance at the soft palate level Comparison of airway clearance at the soft palate level between Group SS and Group S during oral fiberoptic-guided intubation. Data are presented as frequency (n) and percentage (%). A statistically significant difference was observed between the two groups (χ²(2)=16.26, p<0.001), where 2 is the degree of freedom. Individual p-values for no obstruction, partial obstruction, and complete obstruction were 1.000, 0.006, and 0.001, respectively. The chi-square test was used for overall comparison.

Airway Clearance at Soft Palate	Group SS (n=30)		Group S (n=30)		
	Frequency (n)	Percentage (%)	Frequency (n)	Percentage (%)	P-value
No obstruction	13	43.3	13	43.3	1.000
Partial obstruction	17	56.7	6	20.0	0.006
Complete obstruction	0	0.0	11	36.7	0.001
Total	30	100	30	100	<0.001

In contrast, airway clearance at the epiglottis level was comparable between Group SS and Group S (Table 2).

**Table 2:** Airway clearance at the epiglottis level Comparison of airway clearance at the epiglottis level between Group SS and Group S during oral fiberoptic-guided intubation. Data are presented as frequency (n) and percentage (%). No statistically significant difference was observed between the two groups (χ²(1)=2.70, p=0.100), where 1 is the degree of freedom. The chi-square test was used for statistical analysis. A p-value could not be calculated for complete obstruction, as no events were observed in either group.

Airway Clearance at Epiglottis	Group SS (n=30)		Group S (n=30)		P-value
	Frequency (n)	Frequency (n)	Percentage (%)	Percentage (%)	
No obstruction	23	17	56.7	76.7	0.115
Partial obstruction	7	12	43.3	23.3	0.115
Complete obstruction	0	0	0.0	0.0	N/A
Total	30	30	100	100	0.100

The time taken to visualize the vocal cords and carina was significantly shorter in Group SS compared to Group S. However, the time required for advancement of the endotracheal tube was comparable between the two groups (Table 3).

**Table 3:** Time taken to view vocal cords, carina, and tube advancement Comparison of time to visualize the vocal cords, time to visualize the carina, and time for endotracheal tube advancement between Group SS and Group S during oral fiberoptic-guided intubation. Data are expressed as mean±SD, median, and range. An independent samples t-test was used for statistical analysis.

Time (sec)	Group SS (n=30)			Group S (n=30)			P-value
	Mean±SD	Median	Min-Max	Mean±SD	Median	Min-Max	
Time to visualize vocal cords	12.35±6.68	12.00	4.4-26.0	15.99±5.37	15.48	6.4-26.6	0.024
Time to visualize carina	19.92±7.10	19.03	10-36	25.05±7.03	23.86	12-37	0.007
Time for tube advancement	10.92±6.69	8.85	4.0-29.0	9.32±5.21	7.90	4.0-24.5	0.303

The number of attempts required for endotracheal tube advancement was comparable between Group SS and Group S, with no statistically significant difference observed between the two groups (Table 4).

**Table 4:** Attempts made for tube advancement Comparison of the number of attempts required for endotracheal tube advancement between Group SS and Group S during oral fiberoptic-guided intubation. Data are presented as frequency (n) and percentage (%). No statistically significant difference was observed between the two groups (χ²(2)=2.08, p=0.353), where 2 is the degree of freedom. The chi-square test was used for overall comparison.

Attempts for Tube Advancement	Group SS (n=30)		Group S (n=30)		P-value
	Frequency (n)	Percentage (%)	Frequency (n)	Percentage (%)	
1	24	80.0	26	86.7	0.731
2	6	20.0	3	10.0	0.472
3	0	0.0	1	3.3	1.000
Total	30	100	30	100	0.353

As shown in Table 5, the mean time to achieve intubation was comparable between Group SS and Group S, with no statistically significant difference observed between the two groups.

**Table 5:** Mean time to achieve intubation Comparison of the mean time to achieve intubation between Group SS and Group S during oral fiberoptic-guided intubation. Data are expressed as mean±SD, median, and range. No statistically significant difference was observed between the two groups (p=0.095). An independent samples t-test was used for statistical analysis.

Time (s)	Group SS			Group S			P-value
	Mean±SD	Median	Min-max	Mean±SD	Median	Min-max	
Time to achieve intubation	30.85±8.49	28.71	19.1-56.0	34.43±7.82	34.49	21.4-49.0	0.095

As shown in Table 6, signs of trauma at extubation were comparable between Group SS and Group S, with no statistically significant difference observed between the two groups.

**Table 6:** Signs of trauma during extubation Comparison of signs of trauma at extubation between Group SS and Group S during oral fiberoptic-guided intubation. Data are presented as frequency (n) and percentage (%). No statistically significant difference was observed between the two groups (p=1.000). Fisher's exact test was used for statistical analysis.

Sign of Trauma at Extubation	Group SS		Group S		P-value
	Frequency (n)	Percentage (%)	Frequency (n)	Percentage (%)	
No	27	90.0	26	86.7	1.000
Yes	3	10.0	4	13.3	1.000
Total	30	100	30	100	1.000

As shown in Table 7 and Table 8, the incidence of postoperative sore throat at one hour and six hours after extubation was comparable between Group SS and Group S, with no statistically significant difference observed between the two groups.

**Table 7:** Post-extubation sore throat at one hour Comparison of postoperative sore throat at one hour after extubation between Group SS and Group S during oral fiberoptic-guided intubation. Data are presented as frequency (n) and percentage (%). No statistically significant difference was observed between the two groups (p=1.000). Fisher's exact test was used for statistical analysis.

Post-extubation Sore Throat at One Hour	Group SS		Group S		P-value
	Frequency (n)	Percentage (%)	Frequency (n)	Percentage (%)	
No	29	96.7	28	93.3	1.000
Yes	1	3.3	2	6.7	1.000
Total	30	100	30	100	1.000

**Table 8:** Post-extubation sore throat at six hours Comparison of postoperative sore throat at six hours after extubation between Group SS and Group S during oral fibreoptic-guided intubation. Data are presented as frequency (n) and percentage (%). No statistically significant difference was observed between the two groups (p = 0.492). Fisher’s exact test was used for statistical analysis.

Post-extubation Sore Throat at Six Hours	Group SS		Group S		P-value
	Frequency (n)	Percentage (%)	Frequency (n)	Percentage (%)	
No	30	100	28	93.3	0.492
Yes	0	0	2	6.7	0.492
Total	30	100	30	100	0.492

As shown in Table 9 and Table 10, postoperative hoarseness of voice was minimal in both groups. Only one patient (3.3%) in Group SS had hoarseness of voice at six hours after extubation, while no patient in Group S experienced hoarseness at any time after extubation.

**Table 9:** Post-extubation hoarseness at one hour Comparison of postoperative hoarseness at one hour after extubation between Group SS and Group S during oral fiberoptic-guided intubation. Data are presented as frequency (n) and percentage (%). Statistical analysis could not be performed because no events were observed in either group. Fisher's exact test was intended for statistical analysis.

Post-extubation Hoarseness at One Hour	Group SS		Group S		P-value
	Frequency (n)	Percentage (%)	Frequency (n)	Percentage (%)	
No	30	100.0	30	100.0	N/A
Yes	0	0.0	0	0.0	N/A
Total	30	100	30	100	N/A

**Table 10:** Post-extubation hoarseness at six hours Comparison of postoperative hoarseness at six hours after extubation between Group SS and Group S during oral fiberoptic-guided intubation. Data are presented as frequency (n) and percentage (%). No statistically significant difference was observed between the two groups (p=1.000). Fisher's exact test was used for statistical analysis.

Post-extubation Hoarseness at Six Hours	Group SS		Group S		P-value
	Frequency (n)	Percentage (%)	Frequency (n)	Percentage (%)	
No	29	96.7	30	100.0	1.000
Yes	1	3.3	0	0.0	1.000
Total	30	100	30	100	1.000

## Discussion

Maintenance of upper airway patency during anesthesia is essential because anesthesia reduces neuromuscular support of the pharyngeal airway, increasing the tendency for airway narrowing [3-5]. Under these conditions, airway patency becomes increasingly dependent on structural airway mechanics. Backward movement of upper airway soft tissues may impair visualization during fiberoptic-guided intubation and make airway manipulation more difficult [3,4]. Various maneuvers, including jaw thrust, chin lift, lingual traction, and positional modifications, have therefore been used to improve airway patency during fiberoptic-guided intubation [6-10].

Flexible bronchoscopy allows indirect visualization of the laryngeal inlet and is widely used when direct laryngoscopy is difficult or undesirable. Adequate airway patency is essential for successful fiberscopy [3-5]. Previous studies have demonstrated that body elevation improves upper airway patency during sedation and anesthesia. Ikeda et al. reported that 30° body elevation reduced upper airway collapsibility during midazolam sedation, while Tagaito et al. demonstrated reduced pharyngeal collapsibility in anesthetized patients positioned sitting rather than supine [11,12]. Chang et al. also observed improved laryngeal visualization in a 25° semi-sitting position [13].

In the present study, we evaluated the effect of a 25° semi-sitting position on airway clearance achieved with jaw thrust during oral fiberoptic-guided intubation. We found significantly better airway clearance at the soft palate level in the semi-sitting group compared with the supine group (p<0.001). In addition, the time required to visualize the vocal cords (p=0.024) and carina (p=0.007) was significantly shorter in the semi-sitting group. However, airway clearance at the epiglottis level, time for tube advancement, number of attempts for tube advancement, and total intubation time were comparable between the two groups.

Airway clearance at the soft palate level was assessed by observing contact between the soft palate or uvula and the dorsum of the tongue, while airway clearance at the epiglottis level was assessed by observing contact between the epiglottis and the posterior pharyngeal wall. Nandi et al. previously demonstrated that the soft palate is the principal site of upper airway obstruction during anesthesia, with a lesser contribution from the epiglottis [3]. Accordingly, airway clearance at the soft palate level was selected as the primary outcome in our study.

Our findings demonstrated significantly better airway clearance at the soft palate level in the semi-sitting group compared with the supine group, whereas airway clearance at the epiglottis level was similar between groups. Complete soft palate obstruction occurred only in the supine group, while no patient in the semi-sitting group developed complete obstruction. At the epiglottis level, no complete obstruction was observed in either group. These findings are consistent with those reported by Chang et al., who also demonstrated improved airway clearance at the soft palate level in the semi-sitting position [13]. Although the overall airway clearance observed in our study was lower than that reported previously, the relative differences between groups were comparable. This variation may have been related to differences in the degree of jaw thrust applied despite standardization of the technique.

Flexible bronchoscopic intubation may require longer airway instrumentation time than conventional laryngoscopy [14-16]. This potentially increases the risk of hypoxemia and hemodynamic stress responses. In our study, the time required to visualize both the vocal cords and carina was significantly shorter in the semi-sitting group. These findings are likely related to improved airway clearance at the soft palate level, facilitating smoother passage of the fiberscope.

The time required for advancement of the endotracheal tube over the fiberscope was comparable between groups, and the first-attempt success rate for tube advancement was similar in both positions. Minor difficulty during tube passage was managed by slight withdrawal and 90° anticlockwise rotation of the tube before re-advancement. These findings suggest that patient positioning mainly influences airway visualization rather than the mechanics of endotracheal tube advancement.

Although the total intubation time was shorter in the semi-sitting group, the difference did not reach statistical significance (p=0.095). This may have been related to the relatively small sample size and the time-bound nature of the study.

Airway trauma and postoperative airway symptoms were minimal in both groups. Blood staining on the endotracheal tube during extubation, postoperative sore throat, and postoperative hoarseness were infrequent and comparable between groups. No patient required rescue intubation by direct laryngoscopy, and no episodes of significant desaturation occurred during the procedure.

Our study had several limitations. Blinding of the anesthesiologist performing fiberoptic-guided intubation was not possible because patient positioning was evident during the procedure. The sample size was relatively small because of the time-bound design of the study. Although clinically relevant differences in total intubation time were observed, a larger study may have demonstrated statistical significance.

## Conclusions

In conclusion, jaw thrust in the 25° semi-sitting position provides superior airway clearance at the soft palate and allows faster visualization of the vocal cords and carina during oral fiberoptic intubation. Semi-sitting positioning may therefore be a useful adjunct to improve pharyngeal patency and facilitate fiberoptic-guided intubation in anesthetized patients. However, airway clearance at the epiglottis level, time for tube advancement, number of tube advancement attempts, and total intubation time were comparable between the semi-sitting and supine positions. The incidence of airway trauma, postoperative sore throat, and postoperative hoarseness was low and similar in both groups.
